# Supplementary material for: Quantifying online citizen science: Dynamics and demographics of public participation in science
Source: PLoS One. 2023 Nov 21;18(11):e0293289. doi: 10.1371/journal.pone.0293289 (PMC10662724; doi:10.1371/journal.pone.0293289)
Supplement: Attachment — Submitted filename: Response_to_Reviewers_Strasser.pdf [file pone.0293289.s002.pdf]

Replies to Reviewers

Reviewer #1: The authors examined the global demographics of participation in citizen science projects were contributions can be provided online. They analysed the large amount of about 14 million participants worldwide (and not only in „Western“ countries) aiming to understand whether citizen science can contribute to the democratization of science. They concluded that due to the – in fact typical – overrepresentation of more science affine persons and the uneven distribution of participation (which is also typical for each social group ranging from sport associations to parents evenings at schools) online citizen science projects provide only a limited contribution to the democratization of science understood as the increasing participation of underrepresented groups.

Their observation of a peak on 2000 and the general timely dynamic is also interesting and worth thinking about a bit more. I may be linked to the general desire for more real social contacts, or a change in citizens scientists expectations but also technical opportunities.

The study is very interesting and scientifically sound. The database is disciplinarily and geographically mixed. The study provides hints how to monitor citizen science activities during a given time period – and therefore a tool to understand better the different interaction levels. I may also help to see social targets such as democratization or increasing scientific literacy a bit more differentiated.

Or to put it the other way round: State investments into education may also increase the value participants can gain from citizen science.

A learning point for citizen science project manager is that the „platformisation“ may help to keep participants with fluctuating interests

From my perspective the authors miss an important point in their discussion. Mainly data from online participation are used for machine learning which implies that the role of citizens and therefore their motivations may change, but also that the shown uneven distribution of contributors may impact the algorithms.

Response: *Thanks! We already mentioned the impact of AI in the discussion but expanded our remarks along the suggested lines: “The growing integration of AI in citizen science is already transforming the field.*

Since citizen science data is now used for AI training, the results presented here draw attention to the potential biases introduced by the uneven distribution of participant demographics and the necessity to monitor the profiles of participants (Loftian 2021). In the near future, AI could also reduce significantly the need for citizen scientists. Since AI can perform some of the tasks more efficiently than humans, it could impact the historical trends described in this paper, especially for tasks such as analysis."

Reviewer #2: The authors present a study with important goals – to understand participation in "participatory" science, and they assembled an especially large database of participants from three types of projects: computing (providing computational power from their personal computers), analyzing (categorizing data or attempting to solve scientific problems), and sensing (reporting biodiversity observations). The authors presented several metrics to gauge participation across project types with a particular focus on gender, age, and country participation.

I found it very difficult to evaluate this research for two reasons, and so have declined to give it a full review. First, there was no place where they actually explained each of the projects (e.g., what is "BIOINC" – what is the difference between the different programs, e.g., SETI at home vs. climate, what is "FOLD-it" or the Multi-game universe". I am familiar with some of these, but not all – and the authors should describe each). A table with every single project from which they extracted data would be extremely useful, including start and stop years.

Response: We added a description of each project considered in this paper, except for the ten BOINC projects which all operate in the same way, but with a different goal. Table 1 lists all the projects with their starting date, but not ending date, since they are all still active (except SETI@home).

I also was not able to see results in all figures (especially Fig. 5) in that the graphic was so fuzzy, I couldn't read the labels. Following the results was also made difficult because figures were at the end of the manuscript, while figure labels were dispersed throughout. I couldn't keep hunting around for figure legends.

Response: This must be a technical problem on the PLOS platform, as the figures we uploaded are all in high resolution.

So, even though I am not providing a formal review, I do have some suggestions for the authors:

- Give more consideration to how you evaluate "computing" and other activities. Is offering computational resources to science being a citizen scientist? Or is it just donating resources? Are those resources even needed anymore? It seems that by 2017 (last year of data as per Fig. 3), participation was very low- and by 2021, only 0.1%. Does it matter that most of the people providing computational resources in the early 2000s were male and in rich countries (where it is more likely that a private citizen would have a computer?). Yet because these programs engaged so many more people, they appear to swamp out other types of participation. Participation that is, in my opinion, more relevant to "citizen" or participatory science.

Response: This comment raises two important points, also raised by other reviewers.

1. Does distributed computing quality as citizen science?

   See Comment 1 to the Editor above.

1. Does the large size of the distributed computing sample lead to unwarranted generalisations?

   See Comment 2 to the Editor above.

- Your methods of designating gender and age seemed dubious at best. It seems safer to use this information only when you have some profile data. Determining gender and age based on user name (although it was not entirely clear that was what was being used – because the methods were not spelled out), seems risky.

Response: We understand the reviewer's skepticism. However, the method is well-established and we have now described it more clearly in the "Gender coding" section and cited the relevant scientific literature. Furthermore, we have validated the method of determining gender though usernames by comparing it to the tedious, but reliable, method of manually determining gender by reading 12,000 profiles. The results were the same for each of the six projects (or 2,000 profiles each). We thus feel confident that the method of assigning probabilistic genders in first names included in usernames is a good approximation of the actual gender of the participants.

Reviewer #3: The manuscript by Bruno Strasser et al. provides a novel and interesting insight into the worldwide development of online CS. Three important goals of CS are critically examined and discussed based on a large data set, thus making an important contribution to a realistic appraisal of online CS.

However, some of the methods, such as automatic coding, should be explained in more detail. It is also important to discuss the extent to which 'distributed computing' can be classified as citizen science. Some of the wording in the results section should also be reviewed, as it reads more like a discussion of the results.

-Abstract: Correct sentence: 'These results highlight current challenges and the future potential of citizen science.'

Response: Corrected, thanks!

-Methods:

-Because automatic coding is a central method in your paper, please introduce it in more detail in the beginning of the methods section, explaining its principles, aims and approaches. Specify the functioning – which package was used, is it AI/ algorithm based?

Response: We now give a more detailed explanation of automatic coding. Automatic coding was performed by identifying potential first names in username strings using a first name databased constructed from census data and assigning a gender probability based on the gender frequency in the database.

-l. 143-144. please discuss to which extent 'computing' i.e. providing computing capacity can be considered as a real CS activity- also taking into account the ten principles of CS (ECSA, 2015). What are participant motivations for computing? What benefits does computing have for participants?

Response: This is an important point and has been addressed in our Comment 1 to the Editor above. However, we do not discuss participant

motivations as they have no bearing on the definition of citizen science nor on the results of this study.

-L. 184: please describe what the platform names.org was used for exactly

Response: We now describe precisely how we did the gender coding. The names.org database was used to assign a gender during the manual coding process.

-L. 187ff: please reformulate this sentence to explain: how can you assign gender to a user profile that doesn't exist?`

Response: Automatic coding is applied to usernames, not profiles. We clarified the text.

-L. 230: please explain why you used Lorenz curves, eg 'to visualize xxx, we used…'

Response: Thanks, we explain this in the text now.

-Fig. 1: Add a legend to briefly explain three forms of participation. In methods part, you use the words: computing, analyzing, observing, in the figures, you write computing, sensing, analyzing- why this difference in terms?

Response: Thanks, that was mistake, *sensing* is the term we use systematically. The text has been corrected.

-L. 287-342: several sentences read like you are already discussing the results in this section, instead of just reporting results in a neutral way, e.g.:

Response: Correct. However, the extent to which a Results section should only present "raw data" and leave all the data interpretation to the discussion section is a matter of debate. Given the length of the paper, and to facilitate its understanding for the reader, we decided to highlight the context of the data in the Results section and discuss the further implications and limitations in the Discussion section.

-Reformulate l.287-288: ‘This result, alone, already challenges the common narrative 287 about the recent “rise of citizen science” [37–39] or “rise of the citizen scientist” as an editorial in Nature put it’

-L 310 ‘misleading’

-L 329-330: ‘This demonstrates that the total number of accounts is a poor indicator of participant activity.’

-L. 341 ‘Hence, it is reasonable to say that there are under one million monthly

participants in citizen science year-round.’ -Response: this belongs into the discussion part.

Response: Thanks, we made all the changes above accordingly.

Moreover, please always make it clear that you are talking about online CS. The considerable offline CS activities worldwide are not included.

Response: This is an important point. We further clarified in the text that we are only talking about *online* citizen science.

-L. 354 ff: you mention the Gini coefficient here, but this wasn’t mentioned or explained in the methods part

Response: We explained and added a referenced in the Methods section.

-L. 398: typo: cicizen science

Response: Thanks!

-L. 415-419: this could fit into the introduction or discussion, rather than in results

Response: Yes, me moved this section to the discussion.

-L.442: specify: ‘online citizen science population’ –you didn’t analyze offline CS activities

Response: Correct. We added “online” here and elsewhere.

-L. 473: ‘as citizen science have valued the participation of ‘ordinary citizens’: specify - do you mean that CS actors or the CS community has valued xxx?

Response: CS project organizers. We clarified the text.

Discussion

-L.606ff: when discussing CS learning outcomes, it doesn’t make sense to talk only about participant numbers or visitor numbers. It is important to differentiate different forms of CS participation or engagement with science (Shirk et al. 2012)- eg computing projects (do they actually offer any learning outcome??) can hardly be compared to real CS activities where people receive training, interact with others and learn to interpret ecological data. An aspect to be considered is that a museum visit is a short-time event, while CS engagement can be a long-term, repeated learning opportunity. It would be helpful to include some of these thoughts into your discussion, also stating clearly that your data don’t comprise any information about learning outcomes

Response: Right, it is important to differentiate between different kinds of science learning and we do so in the discussion (“many of these individuals experience different learning opportunities than participants in citizen science projects, where they may acquire not only scientific content knowledge, but also knowledge about the nature of scientific inquiry”). As for computing projects, studies in science education have shown unambiguously that they offer significant learning outcomes (see the work of L. Kloetzer among others). We added a point about the possibility for long-term engagement in citizen science, even though participation is typically short-lived, and about the absence of data on learning outcomes.

-L. 637: ‘Its value and significance can thus not be fully captured by the number of its practitioners alone.’ -Response: agree completely, could you highlight this sentence more in the abstract and beginning of discussion/ conclusion? It seems quite buried

Response: Right, we added this point directly in the abstract as well as the first paragraph of the discussion.

Reviewer #4: An interesting paper that strives to characterise citizen science as a 'discipline' in its entirety rather than at an individual project level like most other studies. I think this paper will be heavily cited, and I admire the authors for tackling such a huge dataset. I also like that you sought to actually test whether 'entrenched'/much-parroted claims like 'citizen science is on the rise' are actually backed up by the stats rather than just accept it as true like all other studies (even if I don't fully agree with some of your rebuttals to these claims). My comments and suggestions are in the attached word file; most of them are minor corrections of typos or asking for clarification in places where I was confused or unclear about eg methodology, but a few important points here also:

1. I was unable to download or view any of your supplementary material. After clicking the download button, I waited for more than one hour and it did not work, all that happened was an infinite spinning circle over the download button, and every now and then the button would flash and say ‘request access’ instead of download. I also closed the page and tried again multiple times, including in multiple different browsers, but the download failed every time. I also emailed the link to multiple other people, and they couldn't download the material either. Could you double check whether you're able to download those files yourselves from the provided link, or whether the download is broken. It was unfortunate I couldn't access this material as I assume it would have answered some of my queries.

Response: We checked with Yareta, and there was indeed a problem with the download function. It had been corrected and all data is now available for download with the same link and doi.

2. Perhaps my biggest concern is the extreme influence of a single outlier on your entire dataset, ie SETI@home. I completely acknowledge that you can't just omit it from your study because it's skewing results, and you do explicitly note its influence in your MS, **but it seems like many of your major claims and findings are effectively being entirely driven by this**

single project. If you were to remove it, it seems like many of your reported trends/stats would be wildly different, and indeed you do already report this; for example, SETI@home had 90% males vs iNat 56% and eBird 51%, dramatically different figures. Growth peaked over 20 years ago (!!) for SETI@home, but eBird and iNat continue to grow exponentially. Perhaps this is too much additional work/would add too much complexity to your study, but I would be very interested in two parallel sets of results being presented, one with and one without SETI@home.

Response: This concern was voiced by another reviewer and we addressed it carefully (see Comment 2 to the Editor above and response to comment 29 by Reviewer 4). In the manuscript we are now even more careful to distinguish the unique impact of SETI@home from the more general trends in online citizen science.

I think it's also a very relevant fact that as of March 2020, SETI@home has terminated sending new data to the public, and the citizen science aspect of the project has effectively gone into hibernation; you don't mention this at all in your paper, but it's an important point to acknowledge.

Response: Right, we mention this now in the text.

3. As an extension of my above comment, some of the major conclusions and findings that you report are effectively entirely based on the computing category (which of course includes SETI@home). Indeed, your findings for education/profession were based exclusively on computing projects, given you say "The analyzing projects on Zooniverse have no biographical profiles" and that "For sensing projects, such as iNaturalist or eBird, self-descriptions center on the affective dimension of amateur naturalist pursuit, and rarely mention occupation or education, preventing a comparison with the other projects". You do mention and cite earlier surveys on these two fronts, but of course this isn't the same as actually manually coding profiles from these projects like you did for the computing projects.

Response: This is not quite correct. We did not rely exclusively on computing projects (BOINC), since we also analyzed FoldIt profiles manually and discussed the results. Unfortunately, the inclusion of FoldIt

was only mentioned in the results section, and not in the methods question. This has now been corrected,

My worry is that your paper will be prone to 'mis-citations' by other authors that do not carefully read your paper and simply skim the abstract and results/discussion. Of course you have no control over that element and I don't expect you to take any responsibility for those cases, but I think you could perhaps pre-emptively address this issue by including more explicit explanations/caveats than what you currently have, or subtly adjusting some of your wording. For example, your abstract notes that "the vast majority of participants, male and female, have a background in science", but this is, in my opinion, misleading as a statement in isolation given, again, it is based entirely (from the perspective of your specific quantitative analysis) on 12,000 profiles (out of 14 million accounts, =0.09%!!) selected only from the computing category, and literally zero profiles from any of the analyzing or sensing projects.

Response: Right, we have now added a “pre-emptive” comment in the abstract as well and give a more balanced view highlighting the contribution of sensing projects. By the way (see Comment 2 to the Editor above and response to comment 29 below), the result on the scientific background of participants in *not* solely based on BOINC, but also on FoldIt (90 % of the participants have a background in science or IT). We have no data about participant backgrounds in *sensing* projects. However, the literature on the demographics of *sensing* projects (biodiversity monitoring) based on surveys confirms the trends we highlight in our paper (Pateman 2021, Dohmnaill 2020, etc)

As for the sample size 14,000 profiles (12,000 from BOINC + 2,000 from FoldIt), it is greater than any existing study and already represents several weeks of tedious reading and coding by three of the authors (including the lead author). In the text, we draw attention to the fact that our data comes from this limited subset.

Further comments by Reviewer 4

1. Line 44: ‘highlighting’ should be ‘highlight’.

Response: Corrected, thanks!

2. Lines 110-111: At line 91, you state that you use the terms public participation and citizen science interchangeably in your MS. Yet here, stating 'focusing on citizen science' implies that you perceive citizen science to be a subset or type of public participation of science. I have no qualms with either interpretation in and of itself, however, your statements seem to be inconsistent with each other in this context.

Response: Thanks, we corrected this now. The terms are not used interchangeably, since citizen science is a subset of public participation.

3. Lines 120-121: “it is often difficult, if not impossible, to control for sample bias in anonymized surveys” is there a reference for this?

Response: No, it’s a logical point. Some surveys include variables such as sex and age that can be compared to the general population to evaluate if the sample is demographically representative. But the bias is difficult to correct since the most relevant variables, such as the online activity of the responders in the citizen science project, are usually not queried in the surveys.

4. Lines 121-122: “Furthermore, “survey fatigue” among participants hinders further data collection” again, I think a reference would be useful here if one exists.

Response: Thanks, we added Ackland and O’Neal, 2011.

5. Line 134: should “1999 and 2021” instead be ‘...from 1999 through to 2021”?

Response: Corrected, thanks!

6. Line 135: multi multiple

Response: Corrected, thanks!

7. Line 144-146: how did you choose the four analyzing projects and the two observing projects? Are they, similar to the BOINC cases, the largest projects within those two modes? And if yes, why only the top 4 and top 2 versus the top 9 + SETI@home within BOINC?

Response: We took the largest projects in each category provided they had over 100k accounts. We made this clear in the text now.

8. Line 162: was were

Response: Corrected, thanks!

9. Line 171: “the six BOINC projects” which six, given in lines 143-144 you say you looked at nine BOINC projects in addition to SETI@home. The six in parentheses in lines 156-157?

Response: Corrected: Yes, the top six.

10. Lines 175-176: What is this assumption based on? Is this number based on previous statistical analyses, eg a study that found 95% of married couples consist of heterosexual relationships? A brief explanation/reference here would be useful.

Response: Thanks! We re-examined the literature and our data. According to the US Census, there was less than 1% of same-sex married couples in the US during the entire period studied here. We corrected the text and cited the relevant reference. The rounded result presented in the paper remain unchanged due to the small number of cases.

11. Lines 176-177: re gender assignment, how did you deal with gender-neutral names such as Chris or Kim?

Response: Corrected: When gender assignment was done manually, the most probable gender was assigned according to names.org.

12. Line 177: Is the use of this database the norm in studies/methods such as these? (a genuine question, I have no idea). If no, why this database over other options (do they exist?).

Response: Yes, it's a standard method. We added a description of how this method has been used in the literature.

13. Line 178: insert 'by' between for and automatic

Response: Corrected, thanks!

14. Lines 186-187: How? Perhaps this is a naive question, but I don't quite understand how gender information can be extracted when profiles are non-existent.

Response: An error in the text ("profile" instead of "username") made the explanation confusing and has been corrected. 21% of the usernames contain a first name, i.e. bob1972. Most of these names are the first names of the user, as we showed when we calibrated the method with textual profiles. Yes, in some cases it might be the user's cat name. But even in this case, apparently most users will pick a fake name that matches their gender.

15. Lines 187-190: "To determine age and gender across all projects (S0_Profiles_Gender), even those without profiles, and guarantee comparability, we relied on automatic coding for all projects (BOINC, Zooniverse, FoldIt, EteRNA, Eyewire, iNaturalist, eBird)." maybe this would be better at line 212, after you've explained the processes for both age and gender.

Response: Right, since we repeat the point after the "age" section, we deleted the mention of age after the gender section.

16. Line 195: the apostrophe in 2000 should be a comma.

Response: Corrected, thanks!

17. Line 197: did you also search for 'born 20XX'?.

Response: No. But since we exclude users under 10 y.o. (which are mostly likely artefacts), that over 85% of users signed-up before 2010, and that the median age is 35 y.o., we might have missed just a handful of teenagers, at most.

18. Lines 207-208: why would you not calculate it as the difference between the estimated year and 2021? I'm unsure as to why age when they created the account is the most important metric here, rather than say their age when they last used that project. I can understand arguments for both, so I think a brief explanation of why you chose your method would be useful.

Response: We need to estimate the age at the time of profile creation, not the age in year X, since we are comparing two methods to determine the year of birth, one through username, one through profiles. In a profile created in 2010, a user named "bob1972" might say "I'm 38 years old", thus validating the fact that the "1972" in his username is probably his year of birth (and not his zip code or some other number).

19. Lines 209-210: "Because semi-automatic and automatic coding give similar estimates" If they provided similar estimates, why not just use one to avoid redundancy?

Response: To increase the sample size. To plot demographic pyramids, we need age *and* gender. In many cases, only one variable is available.

20. Lines 215-216: not clear to me how these three aspects are relevant to coding for education and occupation, but perhaps that’s my ignorance of studies/methods like these.

Response: Added an example for clarity (“I’m a retired IT sys admin”.)

21. Line 231: “30 days” you mention which specific 30 day period this refers to in line 237 below; I would mention it here instead given this line appears first.

Response: In line 37, we define the period for the active user dataset, not the Lorenz curves. For the Lorenz curves, the period is November 2019, we changed the text accordingly.

22. Line 240: was were.

Response: Yes, data is formally plural, but often used in the singular. We have used the singular throughout the paper.

23. Lines 250-251: In the context of iNaturalist, what do you mean by 'location field'/how did you get these data, given there isn't any one 'field' for this information on users' profiles. There are multiple different ways I could conceive of determining any given user's location on iNat, including:

a. If they're affiliated with one of the nodes, although this isn't foolproof given many countries (including the USA, the largest userbase of any country in the world for iNat) do not belong to nodes, and you aren't forced to be affiliated with a node if you are from that country.

b. Some kind of centroid calculation of all uploaded observations, although again this can be misleading for users who have travelled extensively and made many observations across the world

Response: Neither a. nor b. We received geocodes from the project organizers, who had obtained them from the users’ IP numbers (we did not receive or collect IP numbers). We then coded the countries with GeoPy. We corrected the methods section.

24. Line 254: It is not clear to me what this field actually refers to and why it's important. Lines 255-256 read to me as if you're saying that you define an 'online activity' as the date of account creation. Is this interpretation correct? If asked to define what an online activity is, I would have said the upload of an observation/creation of a data point.

Response: Yes, your interpretation is correct. Ideally, we would define "online activity" at the time when the users produce scientific data/knowledge, i.e. by classifying a galaxy, observing a bird, performing a calculation. However, to ensure comparability across platforms and because there is a lag time between the actual performance of the observation/calculation/analysis and the time stamp when the result is uploaded on the server, we decided to take the time of account creation. We are certain that, at that moment, the user is actually behind his/her computer and the time stamp does not reflect an automatic routine being performed by the computer.

25. Line 261: which sample are you referring to re exclusion of Russia?

Response: The time stamp at the creation of all accounts on BOINC. But the phrasing was awkward, and we clarified.

26. Lines 268-269: I think you need to explicitly clarify here that 14 million have contributed within the 16 specific cit sci projects you analysed. This is very different to saying 14 million in cit sci broadly. Yes, I understand those projects are the 'big players' with a large proportion of total users and are fairly representative of the whole picture, but there are literally thousands and thousands (tens of thousands? hundreds of thousands?) of citizen science projects in addition to your 16. I completely understand it is not feasible whatsoever to analyse them all, but I think it is misleading to make claims about global participation rates and demographics without explicitly noting this caveat, as obvious as it may be.

Response: Agreed. We also added another caveat, namely that we only include "online" projects. However, as discussed later, we think these 16 projects represent a large fraction of all projects, as their number of users

is 3-4 orders of magnitude higher than others cit sci projects. Furthermore, cit sci platforms such as SciStarter had only around 1.5k projects.

UPDATE: reading further I see you explicitly address this multiple times elsewhere in the MS. I still think it would be useful to also do so at this section too.

Response: Yes (see above), we changed the text.

27. Line 145: you refer to iNat and eBird here as belonging to the category “observing”. In Line 269, you also explicitly refer to participants in this category as “observers”, and there are numerous cases throughout where you refer to datapoints as “observations”. However, after that initial use of “observing”, you refer to this same category as “sensing” (eg Line 281, 283, 303). Please change those first uses of observing and observers to sensing/sensors for consistency.

Response: Yes, sorry for the inconsistency, it has now been corrected. However, to be clear, *sensing* is a category, “observations” are one kind of task that we classify under the *sensing* category.

28. Lines 284-285: why are the data missing in that time period?

Response: We completed the analysis in 2019 and stopped monitoring the “last 30 days signup” reports from BOINC. However, finalising this paper took longer than expected and we updated the most recent data at the time of writing in 2021. For eBird and Galaxyzoo, we were also unable to complete retroactively the missing data. In the figure and text, we make clear that there is a temporal gap.

29. Lines 268-269: You refer here to over 14 million **people** having contributed to citizen science. However, the y-axis of Figure 1 is labelled as **accounts**, not people. So the statement in Lines 268-269 should ideally be more nuanced (I’m unsure of the exact wording you want to use); whilst the true number of individual contributors will obviously still be in the same ballpark, there is nonetheless a non-negligible difference between no. of people and no. of accounts. For example, I know of many people who use

both eBird and iNat; here, they are being counted as two different people. I acknowledge the ‘true’ figure is impossible to actually calculate, but I would appreciate that the issue is at least explicitly noted here.

UPDATE: reading further, I see that you now address this issue in Lines 332-342, so feel free to disregard my comment.

Response: Right, but your initial reaction prompted us to add a clarification (“as estimated by the number of user accounts”).

29. Lines 287-289: In my opinion this is an unfair and misleading characterisation of the situation. Your challenge to the common narrative is effectively based entirely on the influence of a single project, SETI@home. Your statement that “Online citizen science was growing faster two decades ago than it does today” is certainly true based strictly on your data, but if you were to remove SETI@home, which is clearly an extraordinary outlier, would this claim still be true? The answer would likely be no, and the ‘new’ results would support the current common narrative (assuming the sensing category would now have the strongest weight).

I also believe that, when referring to the rise of citizen science/scientists, those references that you’ve cited are referring not only to an increase in number of participants, but also a dramatic increase in the number and diversity of projects. Go back to 1999, and it is literally one choice, SETI@home; now, cit sci projects exist for almost every situation imaginable, including collecting data on your baby’s smile or the intelligence of your dog according to Toerpe 2013. I do not have hard evidence to support this claim, but my educated guess is that SETI@home experienced such extraordinary initial growth, that as you state (and I strongly agree with) will almost certainly never be matched again, in large part because it was so novel. As you say, this was really the birth of online cit sci, so the novelty would have attracted many users. Nowadays, the cit sci ‘market’ is massively saturated with so many different projects, many of them subtle variations on the exact same theme, that you’ll never get that same growth because participation/account creation will be distributed across the different projects. But if you combine all the projects together, you do get a sort of analogous growth.

So in summary here, I don't fully support your claim that the common narrative is being challenged, because that challenge seems to be based on the overwhelming influence of a single project that experienced incredible growth under unique circumstances, ie at its inception it was the only online cit sci project that existed. The same applies for your overall characterisation of the computing category in Lines 293-294, because again those numbers are being grossly skewed by SETI@home.

Response: Point taken. See Comment 2 to Editor above. If we exclude SETI@home and all of BOINC (a big "if"), the number of *active* users in cit sci and the number of different projects are both increasing. But… 1/ as you say, "the cit sci 'market' is massively saturated". It seems that the new projects on Zooniverse are tapping into an existing userbase, not expanding the number of people engaged in cit sci. 2/ Only sensing projects, like eBird and iNaturalist still seem to have a sustained growth, as we point out. 3/ These projects happen to represent—and it's no accident—the traditional forms of public participation in science, with the newer forms (analyzing and calculating) being stable or declining. 4/ The claims of "millions" (plural) of participants in individual cit sci projects is grossly exaggerated by 2-3 orders of magnitude. Only SETI reached these magnitudes. So yes, there is still "a rise of citizen science", but today's growth, fueled by eBird and iNaturalist, does not (yet) match the unique explosion of participation created by SETI@home a quarter century ago. To answer your concerns, we added "But this peak in public participation is largely due to a single project with an unmatched number of users: SETI@home. For this reason, we analyzed further the different modes of public participation in science" and later "In the history of online citizen science, these sensing projects are fueling a *second* growth period, after the initial rise due to SETI@home around year 2000."

30. Lines 312-313: (I apologize if this information is somewhere in your supplementary material) Did you define a 'task' in iNat as making an observation? Or did you also include an identification as a task? If the former, then the latter should definitely be included as well. This would not only increase the number of tasks performed, but also increase the number of users completing at least a single task given some users only make IDs without also uploading observations.

Response: Thanks! “Task” has now been explicitly defined in the Materials and Method. It includes observations *only*, and neither identifications, nor comments. When we report the results of the number of users who have performed a single task, we now draw attention to the fact that this reflects only observations. We could have included identifications and comments in our dataset, but in the end decided against it for the following reasons. iNaturalist is really three projects in one: a *sensing* project, where volunteers provide observations, and an *analysing* project, where users provide identifications and/or comments. Because one of the aims of our paper is to compare and contrast the dynamics of different modes of citizen science, we did not want to aggregate tasks belonging to different modes. However, it would indeed be interesting to compare the online behaviors of iNaturalist depending on the kind of contributions (observations, identifications, comments).

31. Table 1: why lump SETI@home in with the other 9 projects on BOINC? Indeed, why lump all the BOINC projects in together at all rather than list them separately

Response: For the sake of clarity and because the numbers are similar for the different BOINC projects, and the breakdown can be found in the supplementary material.

32. Lines 339-340: “Participation to nature observation projects (eBird and iNaturalist) **shows** seasonal variations”

Response: Corrected, thanks!

33. Lines 361-362: regarding division of labour with iNat observations, see https://doi.org/10.1093/biosci/biab093 for a useful reference to cite. There is also an upcoming paper that should be published soon looking at the same concepts, but for identifiers instead. I also want to reiterate here my question from comment 30 above as to what you defined work/tasks as within iNaturalist, given there are two types, observations and identifications, and I worry you only included the former. If you did include both that is fantastic and you can disregard this comment.

Response: We added that useful reference, thanks. For the identifications, see response to comment 30 above.

34. Figure 3: Maybe I'm just being slow, but I find Figure 3 confusing. Why are there multiple blue and red strata, instead of a single red stratum and a single blue stratum that change in height over time?

Response: Yes, it's a hard figure and we struggled to make it clear. Each strata represents a different "cohort", i.e. a population of users who signed-up in a given year. We now adjusted the figure, with a unique color for each strata. It should be less confusing (and makes a nice rainbow).

35. Line 398: cicizen citizen

Response: Corrected, thanks!

36. Line 435: Maybe I'm being ignorant here, but I've never seen a large number written like this before, ie where digits are separated with apostrophes instead of commas or spaces. Is this a standard/accepted convention? Same as my comment no. 16 above.

Response: My wrong, sorry. In French, thousands are separated by an apostrophe (or a space) instead of a comma (in English), and decimals by a coma instead of a period (in English)…

37. Lines 436-441: I would not qualify a close to 50% male/female split for sensing as "unambiguously… male dominated".

Response: Correct, we deleted "sensing" from the list.

38. Lines 444-445: Re a comparison between the scientific workforce and sensing projects, the population has **already** become 'more equal'; you note in Lines 419-420 that the scientific workforce is 40% female, yet your stats in Line 441 indicate 44% females (56% males), ie already closer to the 51% females making up the total population. So for sensing, your

statement should explicitly be that the cit sci population already reflects greater gender equality compared to the scientific workforce.

Response: Correct, we now excluded *sensing* from the general claim, and highlighted the role of sensing projects in reaching gender parity.

39. Lines 527-539: I'm not sold that signup time is an accurate reflection of/proxy for use time. I could easily create an account for a project during my lunch break at work, and then never actually use/participate in that project during work hours. I don't really see any value in this metric/ dimension of your analysis.

Response: Signup time is indeed a proxy, and we use it for the reasons stated above: it is the only comparable and reliable metric we have for human online activity. Yes, people could theoretically create an account during their lunch break and then only participate at night, but that's unlikely (or infrequent). Furthermore, we do not claim that the signup time perfectly reflects the different participatory activities, only that sign-up time shows a measurable time pattern. Most likely, the other activities show a similar pattern, but we leave that to others to explore.

40. In Lines 222-227, you note that you "coded manually the same random samples of 2,000 profiles for each of the six BOINC projects". In Line 469, however, you state "our analysis of profiles of participants across all computing projects". For occupation/education, you did not analyse **all** computing projects, of which there were 10 (SETI@home + 9 others, as stated in Lines 143-144).

Response: Correct, thanks for spotting this. The occupation analysis was indeed performed on the 6 largest BOINC projects, not all computing projects. We made the correction in the text.

41. Lines 569-573: Is this not just an issue of OPAL having a different definition of 'participant' to you? Of course it's fine to exclude them from your analysis given your explicit definition of what a participant is, but if there is not a universal, standardised definition of what a 'participant' is in

a citizen science context, I don't really think you can criticise or devalue their claim in general terms, which is the sense that I get from the somewhat dismissive tone in "they claim to".

Response: Right, we did not mean to criticise OPAL, which is a fantastic project. However, as you noticed, we have been puzzled by the inflation of misleading quantitative claims and by the "dumbing down" of the production of scientific knowledge in some citizen science projects. According to standard definition, such as ECSA's ten principles, to be called "citizen science", a project should produce scientific knowledge, not only share existing knowledge. We changed the phrasing in the text to be more neutral and added about OPAL "because their participant numbers rest on a different definition from ours…".

42. Lines 573-575: you state that "the estimates of… "millions of people" engaged in "a single project" [63] constitute an overestimate", and cite Cooper et al. 2021 [63] as the reference where this claim came from. If I go to that paper, they in turn cite Waldispühl et al. 2020 (https://www.nature.com/articles/s41587-020-0694-x) as the source of the claim. They state that "In fewer than three months, Borderlands Science reached more than 1 million active participants who solved at least one open task". The claim of millions of people engaged in a single project therefore seems completely legitimate to me in this specific case.

Response: Yes, Borderlands Science reached (briefly) "more than 1 million" (singular), but that's still far from the "millions" (plural) figure which is thrown around for projects much smaller than Borderlands Science.

UPDATE: reading further, I see that you explicitly talk about this exact case in Lines 645-651, with the claim that the 1 million plus participants were drawn from an **existing** userbase, and that "participants joined Borderland 3 to play an exciting game, not to solve scientific puzzles." My question here is, are these caveats relevant? If the participants nonetheless engaged in citizen science, regardless of their original intentions, is that valued less than other contributions? To some degree, yes, I do agree it is a 'less significant/important' contribution, especially within the context of your specific analyses. But surely at least some percentage of these

participants learnt something and/or were inspired to contribute to other cit sci efforts due to this experience, so I would not completely dismiss cases such as these, and I think they do hold value.

Response: Exactly. Borderland Science is an exciting new way to reach participants, some of which may be inspired by their experience with citizen science. The point was only that because the incentives (internal to the game) for participation in the science project are so great, the number of participants does not give any indication of a motivation for (citizen) science *per se*. To make sure this comment is not read as a critique of Borderland Science (a great project), we added "(which doesn't mean that participants in Borderland 3 do not make a contribution to citizen science or have a significant science learning experience)".

43. Line 587: do you have a source/reference to support this claim? This will vary wildly among projects. Eg many iNaturalist users spend an order of magnitude more than this per week on the project. Worth briefly noting I think.

Response: Agreed, it will vary widely, and since surveys are mostly answered by highly committed participants, it will be hard to know the actual median participation time. However, the Lorenz curves show the very unequal distribution of work in all projects. There are certainly iNaturalist users who spend dozens of hours per week (or more), but others who contribute only a few minutes every month. We stand by a "few hours" as a reasonable (and generous) estimate of the median contribution across all online projects. It would indeed be interesting to compare iNaturalist and Galaxyzoo for example.

44. Line 706 -Response: omit the space between 20 and %.

Response: This is a very contentious issue, with the Chicago Manual of Style prescribing *not* to use a space, and the SI and ISO standards insisting that a space is *required* ;). We will leave this to the typesetter (as long as it is consistent).
