# Supplementary material for: Quantifying online citizen science: Dynamics and demographics of public participation in science
Source: PLoS One. 2023 Nov 21;18(11):e0293289. doi: 10.1371/journal.pone.0293289 (PMC10662724; doi:10.1371/journal.pone.0293289)
Supplement: Attachment — Submitted filename: review comments.docx [file pone.0293289.s001.docx]

1. Line 44: ‘highlighting’ should be ‘highlight’

2. Lines 110-111: At line 91, you state that you use the terms public participation and citizen science interchangeably in your MS. Yet here, stating 'focusing on citizen science' implies that you perceive citizen science to be a subset or type of public participation of science. I have no qualms with either interpretation in and of itself, however, your statements seem to be inconsistent with each other in this context.

3. Lines 120-121: “it is often difficult, if not impossible, to control for sample bias in anonymized surveys” 🡪 is there a reference for this?

4. Lines 121-122: “Furthermore, “survey fatigue” among participants hinders further data collection” 🡪 again, I think a reference would be useful here if one exists.

5. Line 134: should “1999 and 2021” instead be ‘...from 1999 through to 2021”?

6. Line 135: multi 🡪 multiple

7. Line 144-146: how did you choose the four analyzing projects and the two observing projects? Are they, similar to the BOINC cases, the largest projects within those two modes? And if yes, why only the top 4 and top 2 versus the top 9 + SETI@home within BOINC?

8. Line 162: was 🡪 were

9. Line 171: “the six BOINC projects” 🡪 which six, given in lines 143-144 you say you looked at nine BOINC projects in addition to SETI@home. The six in parentheses in lines 156-157?

10. Lines 175-176: What is this assumption based on? Is this number based on previous statistical analyses, eg a study that found 95% of married couples consist of heterosexual relationships? A brief explanation/reference here would be useful.

11. Lines 176-177: re gender assignment, how did you deal with gender-neutral names such as Chris or Kim?

12. Line 177: Is the use of this database the norm in studies/methods such as these? (a genuine question, I have no idea). If no, why this database over other options (do they exist?)

13. Line 178: insert ‘by’ between for and automatic

14. Lines 186-187: How? Perhaps this is a naive question, but I don't quite understand how gender information can be extracted when profiles are non-existent.

15. Lines 187-190: “To determine age and gender across all projects (S0_Profiles_Gender), even those without profiles, and guarantee comparability, we relied on automatic coding for all projects (BOINC, Zooniverse, FoldIt, EteRNA, Eyewire, iNaturalist, eBird).” 🡪 maybe this would be better at line 212, after you've explained the processes for both age and gender

16. Line 195: the apostrophe in 2000 should be a comma

17. Line 197: did you also search for 'born 20XX'?

18. Lines 207-208: why would you not calculate it as the difference between the estimated year and 2021? I’m unsure as to why age when they created the account is the most important metric here, rather than say their age when they last used that project. I can understand arguments for both, so I think a brief explanation of why you chose your method would be useful.

19. Lines 209-210: “Because semi-automatic and automatic coding give similar estimates” 🡪 If they provided similar estimates, why not just use one to avoid redundancy?

20. Lines 215-216: not clear to me how these three aspects are relevant to coding for education and occupation, but perhaps that’s my ignorance of studies/methods like these.

21. Line 231: “30 days” 🡪 you mention which specific 30 day period this refers to in line 237 below; I would mention it here instead given this line appears first

22. Line 240: was 🡪 were

23. Lines 250-251: In the context of iNaturalist, what do you mean by 'location field'/how did you get these data, given there isn't any one 'field' for this information on users' profiles. There are multiple different ways I could conceive of determining any given user's location on iNat, including:

a. If they're affiliated with one of the nodes, although this isn't foolproof given many countries (including the USA, the largest userbase of any country in the world for iNat) do not belong to nodes, and you aren't forced to be affiliated with a node if you are from that country.

b. Some kind of centroid calculation of all uploaded observations, although again this can be misleading for users who have travelled extensively and made many observations across the world

24. Line 254: It is not clear to me what this field actually refers to and why it's important. Lines 255-256 read to me as if you're saying that you define an 'online activity' as the date of account creation. Is this interpretation correct? If asked to define what an online activity is, I would have said the upload of an observation/creation of a data point.

25. Line 261: which sample are you referring to re exclusion of Russia?

26. Lines 268-269: I think you need to explicitly clarify here that 14 million have contributed **within the 16 specific cit sci projects you analysed**. This is very different to saying 14 million in cit sci broadly. Yes, I understand those projects are the 'big players' with a large proportion of total users and are fairly representative of the whole picture, but there are literally thousands and thousands (tens of thousands? hundreds of thousands?) of citizen science projects in addition to your 16. I completely understand it is not feasible whatsoever to analyse them all, but I think it is misleading to make claims about global participation rates and demographics without explicitly noting this caveat, as obvious as it may be.

UPDATE: reading further I see you explicitly address this multiple times elsewhere in the MS. I still think it would be useful to also do so at this section too.

27. Line 145: you refer to iNat and eBird here as belonging to the category “observing”. In Line 269, you also explicitly refer to participants in this category as “observers”, and there are numerous cases throughout where you refer to datapoints as “observations”. However, after that initial use of “observing”, you refer to this same category as “sensing” (eg Line 281, 283, 303). Please change those first uses of observing and observers to sensing/sensors for consistency.

28. Lines 284-285: why are the data missing in that time period?

29. Lines 268-269: You refer here to over 14 million **people** having contributed to citizen science. However, the y-axis of Figure 1 is labelled as **accounts**, not people. So the statement in Lines 268-269 should ideally be more nuanced (I’m unsure of the exact wording you want to use); whilst the true number of individual contributors will obviously still be in the same ballpark, there is nonetheless a non-negligible difference between no. of people and no. of accounts. For example, I know of many people who use both eBird and iNat; here, they are being counted as two different people. I acknowledge the ‘true’ figure is impossible to actually calculate, but I would appreciate that the issue is at least explicitly noted here.

UPDATE: reading further, I see that you now address this issue in Lines 332-342, so feel free to disregard my comment.

29. Lines 287-289: In my opinion this is an unfair and misleading characterisation of the situation. Your challenge to the common narrative is effectively based entirely on the influence of a single project, SETI@home. Your statement that “Online citizen science was growing faster two decades ago than it does today” is certainly true based strictly on your data, but if you were to remove SETI@home, which is clearly an extraordinary outlier, would this claim still be true? The answer would likely be no, and the ‘new’ results would support the current common narrative (assuming the sensing category would now have the strongest weight).

I also believe that, when referring to the rise of citizen science/scientists, those references that you’ve cited are referring not only to an increase in number of participants, but also a dramatic increase in the **number and diversity of projects**. Go back to 1999, and it is literally one choice, SETI@home; now, cit sci projects exist for almost every situation imaginable, including collecting data on your baby’s smile or the intelligence of your dog according to Toerpe 2013. I do not have hard evidence to support this claim, but my educated guess is that SETI@home experienced such extraordinary initial growth, that as you state (and I strongly agree with) will almost certainly never be matched again, in large part because it was so novel. As you say, this was really the birth of online cit sci, so the novelty would have attracted many users. Nowadays, the cit sci ‘market’ is massively saturated with so many different projects, many of them subtle variations on the exact same theme, that you’ll never get that same growth because participation/account creation will be distributed across the different projects. But if you combine all the projects together, you do get a sort of analogous growth.

So in summary here, I don’t fully support your claim that the common narrative is being challenged, because that challenge seems to be based on the overwhelming influence of a single project that experienced incredible growth under unique circumstances, ie at its inception it was the only online cit sci project that existed. The same applies for your overall characterisation of the computing category in Lines 293-294, because again those numbers are being grossly skewed by SETI@home.

30. Lines 312-313: (I apologise if this information is somewhere in your supplementary material) Did you define a ‘task’ in iNat as making an observation? Or did you also include an identification as a task? If the former, then the latter should definitely be included as well. This would not only increase the number of tasks performed, but also increase the number of users completing at least a single task given some users only make IDs without also uploading observations.

31. Table 1: why lump SETI@home in with the other 9 projects on BOINC? Indeed, why lump all the BOINC projects in together at all rather than list them separately?

32. Lines 339-340: “Participation to nature observation projects (eBird and iNaturalist) **shows** seasonal variations”

33. Lines 361-362: regarding division of labour with iNat observations, see <https://doi.org/10.1093/biosci/biab093> for a useful reference to cite. There is also an upcoming paper that should be published soon looking at the same concepts, but for identifiers instead. I also want to reiterate here my question from comment 30 above as to what you defined work/tasks as within iNaturalist, given there are two types, observations and identifications, and I worry you only included the former. If you did include both that is fantastic and you can disregard this comment.

34. Figure 3: Maybe I’m just being slow, but I find Figure 3 confusing. Why are there multiple blue and red strata, instead of a single red stratum and a single blue stratum that change in height over time?

35. Line 398: cicizen 🡪 citizen

36. Line 435: Maybe I’m being ignorant here, but I’ve never seen a large number written like this before, ie where digits are separated with apostrophes instead of commas or spaces. Is this a standard/accepted convention? Same as my comment no. 16 above.

37. Lines 436-441: I would not qualify a close to 50% male/female split for sensing as “unambiguously… male dominated”.

38. Lines 444-445: Re a comparison between the scientific workforce and sensing projects, the population has **already** become ‘more equal’; you note in Lines 419-420 that the scientific workforce is 40% female, yet your stats in Line 441 indicate 44% females (56% males), ie already closer to the 51% females making up the total population. So for sensing, your statement should explicitly be that the cit sci population already reflects greater gender equality compared to the scientific workforce.

39. Lines 527-539: I’m not sold that signup time is an accurate reflection of/proxy for use time. I could easily create an account for a project during my lunch break at work, and then never actually use/participate in that project during work hours. I don’t really see any value in this metric/dimension of your analysis.

40. In Lines 222-227, you note that you “coded manually the same random samples of 2,000 profiles for each of the six BOINC projects”. In Line 469, however, you state “our analysis of profiles of participants across all computing projects”. For occupation/education, you did not analyse **all** computing projects, of which there were 10 (SETI@home + 9 others, as stated in Lines 143-144).

41. Lines 569-573: Is this not just an issue of OPAL having a different definition of ‘participant’ to you? Of course it’s fine to exclude them from your analysis given your explicit definition of what a participant is, but if there is not a universal, standardised definition of what a ‘participant’ is in a citizen science context, I don’t really think you can criticise or devalue their claim in general terms, which is the sense that I get from the somewhat dismissive tone in “they claim to”.

42. Lines 573-575: you state that “the estimates of… “millions of people” engaged in “a single project” [63] constitute an overestimate”, and cite Cooper et al. 2021 [63] as the reference where this claim came from. If I go to that paper, they in turn cite Waldispühl et al. 2020 (<https://www.nature.com/articles/s41587-020-0694-x>) as the source of the claim. They state that “In fewer than three months, Borderlands Science reached more than 1 million active participants who solved at least one open task”. The claim of millions of people engaged in a single project therefore seems completely legitimate to me in this specific case.

UPDATE: reading further, I see that you explicitly talk about this exact case in Lines 645-651, with the claim that the 1 million plus participants were drawn from an **existing** userbase, and that “participants joined Borderland 3 to play an exciting game, not to solve scientific puzzles.” My question here is, are these caveats relevant? If the participants nonetheless engaged in citizen science, regardless of their original intentions, is that valued less than other contributions? To some degree, yes, I do agree it is a ‘less significant/important’ contribution, especially within the context of your specific analyses. But surely at least some percentage of these participants learnt something and/or were inspired to contribute to other cit sci efforts due to this experience, so I would not completely dismiss cases such as these, and I think they do hold value.

43. Line 587: do you have a source/reference to support this claim? This will vary wildly among projects. Eg many iNaturalist users spend an order of magnitude more than this per week on the project. Worth briefly noting I think.

44. Line 706 -> omit the space between 20 and %
