# Supplementary material for: Quantifying online citizen science: Dynamics and demographics of public participation in science
Source: PLoS One. 2023 Nov 21;18(11):e0293289. doi: 10.1371/journal.pone.0293289 (PMC10662724; doi:10.1371/journal.pone.0293289)
Supplement: Attachment — Submitted filename: review comments - resubmission.docx [file pone.0293289.s003.docx]

1. Line 64: would “who are the participants” possibly be better phrased as “who the participants are”? Not a necessary change, just sounds a bit better to me (especially also since your next item in the sentence uses the same structure as my suggestion).
2. Lines 77-78: “Such approaches are rapidly developing for many online activities, for example on social media,” citation or two useful here
3. Line 120: maybe ‘built’ instead of ‘constituted’? (another personal preference suggestion)
4. Lines 139-153: some inconsistencies with capitalisation here. For the first mention of Computing (Line 139), Analyzing (140) and Sensing (141), you capitalise. At Line 143, you swap computing back to lowercase, and the same for analyzing at Line 150. But at Line 153, you still retain Sensing as capitalised, even when not starting the sentence.
5. Line 168: “…(Amy Robinson), **and** Massively Multiplayer Online Science…”
6. Line 192: citation here for R? (I typically do it for my papers)
7. Line 201: just to make sure I’m totally clear here, so for a username such as ‘thebeachcomber’ (my iNaturalist username), that’s an example where it couldn’t be automatically coded and assigned a gender, correct?
8. Line 255: ‘user id’ should be ‘user ids’ plural?
9. Line 257: ebird url is hyperlinked, remember to do so for others too (although I assume the journal will handle this during proofing)
10. Line 257: should use the term ‘identifications’, not ‘classifications’, for iNaturalist, as identifications is the formal term explicitly used by the platform
11. Line 262: instead of ‘he/she’, which is a little clunky, just use ‘they’ for simplicity?
12. Line 263: missing space between ‘software’ and the citation
13. Lines 321-322: some more inconsistencies here with capitalisation; Sensing is capitalised mid-sentence at Line 322, yet analyzing is lower case at Line 321. Reading further as well, seems like these inconsistencies are found throughout the whole paper, but I didn’t have the time to cross-check whether all of these are only present in the tracked-changes version, or if some inconsistencies are present in both the clean and tracked-changes versions. Please check for this throughout.
14. Line 334: ‘users’ should be ‘user’?
15. Line 336: maybe amend to ‘**active** participants’
16. Line 352: why use the notation ‘k’ for thousand here, rather than just use eg 23,000?
17. Line 356-357: I find this an intriguing conclusion. Is ‘mostly spam’ something you actually assessed, or your hypothesis? Maybe some of the other platforms are skewing the proportion, but from an iNaturalist perspective at least, I would have expected the majority of inactive accounts to have been genuine users who made a small contribution years ago and then left, or who just never contributed for a wide variety of reasons (eg signed up because they attended a workshop but then never used it). As a curator on iNaturalist where one of my roles is suspending users for spam, anecdotally they make up a small % of accounts relative to genuine but inactive users.
18. Line 361: again, I think you should explicitly use the phrase ‘**active** participants’ here, to account for users who may only login once every month or two and thus missed being captured by your June snapshot (even though it’s still probably under 1 million)
19. Lines 375 and 377: just checking, should the Gini values here be 0.84 and 0.82? ie periods instead of commas? Ditto Line 381
20. Line 379: change ‘having’ to ‘have’
21. Line 397: change ‘who’ to ‘which’
22. Line 462: explicitly clarify that these metrics are from the smaller pool that you analysed, not all 14 million?
23. Line 477: 17.20% instead of use of comma
24. Lines 530-531: “have a markedly higher densities of” should be either “have a markedly higher density of” or “have markedly higher densities of”
25. Line 564: unsure if I’m sold on the use of ‘arena’ in this context
26. Line 580: 1,500 still uses an apostrophe here instead of comma
27. Line 590: ‘a safer’, delete the ‘a’
28. Line 655: maybe insert ‘comparatively’ before ‘minor’
29. Line 664: SETI@home began in 1999, not 1990; need to therefore also amend ‘thirty years’ in the next line to ‘twenty years’
30. Line 669: Borderland**s** missing the ‘s’. Needs fixing also in Line 670 and Line 671
31. Line 669: I think ‘which does not mean’ would read much better here as ‘which is not to say’
32. Lines 672-675: this is great, I really like these lines
33. Line 692: I believe Strontium-90 should be hyphenated
34. Line 752: “are actually actively participating **in any given month**”?
